# F/YGG-motif is an intrinsically disordered nucleic-acid binding motif

**DOI:** 10.1080/15476286.2022.2066336

**Published:** 2022-05-01

**Authors:** Joris Van Lindt, Tamas Lazar, Donya Pakravan, Manon Demulder, Attila Meszaros, Ludo Van Den Bosch, Dominique Maes, Peter Tompa

**Affiliations:** a Center for Structural Biology, VIBVIB-VUB, Brussels, Belgium; b Structural Biology Brussels, Vrije Universiteit Brussel, Brussels, Belgium; c Center for Brain & Disease Research, Laboratory of Neurobiology, VIB, Leuven, Belgium; d Department of Neurosciences, Experimental Neurology and Leuven Brain Institute (LBI), KU Leuven, Leuven, Belgium; e Institute of Enzymology, Research Centre for Natural Sciences, Budapest, Hungary

**Keywords:** Liquid-liquid phase separation, disordered proteins, nucleic acid interaction, low complexity domain, sequence motif

## Abstract

Heterogeneous nuclear ribonucleoproteins (hnRNP) function in RNA processing, have RNA-recognition motifs (RRMs) and intrinsically disordered, low-complexity domains (LCDs). While RRMs are drivers of RNA binding, there is only limited knowledge about the RNA interaction by the LCD of some hnRNPs. Here, we show that the LCD of hnRNPA2 interacts with RNA via an embedded Tyr/Gly-rich region which is a disordered RNA-binding motif. RNA binding is maintained upon mutating tyrosine residues to phenylalanines, but abrogated by mutating to alanines, thus we term the RNA-binding region ‘F/YGG motif’. The F/YGG motif can bind a broad range of structured (e.g. tRNA) and disordered (e.g. polyA) RNAs, but not rRNA. As the F/YGG otif can also interact with DNA, we consider it a general nucleic acid-binding motif. hnRNPA2 LCD can form dense droplets, by liquid–liquid phase separation (LLPS). Their formation is inhibited by RNA binding, which is mitigated by salt and 1,6-hexanediol, suggesting that both electrostatic and hydrophobic interactions feature in the F/YGG motif. The D290V mutant also binds RNA, which interferes with both LLPS and aggregation thereof. We found homologous regions in a broad range of RNA- and DNA-binding proteins in the human proteome, suggesting that the F/YGG motif is a general nucleic acid-interaction motif.

## Introduction

The concept of the ‘RNA world’ rests on the idea that early evolution was dominated by RNA molecules capable of genetic information storage as well as its replication via catalytic RNA enzymes, ribozymes [1]. In a major evolutionary transition, RNA was then replaced by DNA for information storage and proteins for catalysis, which infers that RNA–protein interactions provide the most ancient and possibly the most prevalent regulatory mechanism in the cell. In concordance with this idea, there is a very large number of RNA-binding proteins (RBPs) in the proteome: unbiased RNA-interactome capture analyses suggest that there are about 2000 RBPs in the human proteome [2,3], much more than the number of other important protein families, such as kinases (kinome, ~500) [4] or ubiquitin ligases (E3s, ~600) [5], possibly even surpassing in number that of transcription factors (~1600) [6].

The very broad range of protein–RNA interactions encoded by RBPs play key roles in basic RNA-related processes of the cell, such as the assembly of ribosomes, regulation of transcription and mRNA splicing, RNA editing or signal sensing, with such intriguing examples as bacterial immunity relying on the CRISPR/Cas system [3]. Importantly, many RBPs have no direct RNA-related functions, but are implicated in intermediary metabolism, cell-cycle progression, antiviral response, spindle organization or protein metabolism [3,7]. In addition, it is an emerging theme that RNAs may regulate RBP function rather than be regulated by RBPs [3].

Due to diverse functions and regulatory roles, RNA recognition by RBPs has been extensively studied. Most of our insight into the underlying details derives from studying and structurally characterizing specific RNA-binding regions (RNA-binding domains, RBDs) of RBPs. About 17 such ‘canonical’ RBDs, such as RNA-recognition motif (RRM), hnRNP K homology domain (KH), DEAD box helicase domain, double-stranded RNA-binding domain (dsRBD), and cold-shock domain (CSD), are known [2]. In chemical cross link-based direct identification of RBDs (RBDmap), more than 1000 RNA-binding regions have been described [8], half of which lack functional or domain annotations related to RNA biology. Most of these fall into intrinsically disordered regions (IDRs). IDRs are types of protein domains that exist and function without a well-defined 3D structure [9], they often evolve by repeat expansion and harbour repetitive, low-complexity regions [10]. It has been suggested that IDRs may be directly involved in RNA binding [2,11]. Unlike IDRs in general [12], RBP IDRs are noted for their evolutionary sequence conservation, and are suggested to act in RNA binding by the synergy of their internal repeat motifs or cooperativity with adjacent, structured RBDs [13,14]. One of the features of IDRs, in disordered RBDs, is an enrichment in highly repetitive regions, such as Arg-Gly-Gly (RGG) repeats, Arg-Ser (RS) repeats and basic Lys/Arg-rich (K/R) patches [2,14,15]. An additional characteristic IDR, Tyr-Gly or Tyr-Gly-Gly (YG/YGG) repeats has also been noted in RBPs, but while it has not yet been shown directly to bind RNA [3], the YGG motif has been hypothesized as a potential RNA-binding motif in the RBDmap [8]. Recently, it has also been shown to be necessary for proper RNA-chaperone activity of hnRNPD and hnRNPA1 [16] – suggesting it is capable of non-specific RNA interaction, potentially through a similar interaction mechanism as the RGG motif.

A highly exciting field where RNA binding by IDRs and canonical RBD(s), might be of particular relevance is liquid–liquid phase separation (LLPS), in particular by the family of heteronuclear ribonucleoproteins (hnRNPs), such as TDP-43, FUS, hnRNPA1 and hnRNPA2 [17]. These proteins are composed of one or two canonical RBPs (usually RRMs) and one or two long IDRs, also termed low-complexity domains (LCDs) or prion-like domains [18]. hnRNPs are involved in various aspects of RNA metabolism, such as splicing and RNA transport, and they have recently drawn increased attention due to their ability to undergo LLPS [19]. LLPS has been suggested to be the driving force by which liquid-like ribonucleoprotein granules form in the cell. Such granules, also termed membraneless organelles (MLOs), include many well-known structures such as processing bodies and stress granules in the cytoplasm and nucleoli and nuclear speckles in the nucleus [20,21]. MLOs represent a newly recognized general cellular mechanism for the compartmentalization of regulatory pathways and biochemical reactions, without the involvement of confining membranes. In this regard, RNA binding of hnRNPs may be of particular importance, because RNA invariably forms part of MLOs [22] and promotes the LLPS of hnRNPs [23]. RNA can undergo and even drive LLPS, and can recruit proteins to form mature MLOs [24]. Besides RRMs, it has also been suggested that the C-terminal domain (LCD) of another hnRNP (A1) promotes and even directly contributes to RNA and/or DNA binding of the full-length protein [25–27]. The sequence identity of the IDR LCDs of hnRNPA1 and A2 is 72%, which suggests possible functional similarities between the two proteins.

Motivated by these results and the general existence of RNA-binding motifs in IDRs, we have scrutinized hnRNPA2, a protein involved in basic cellular pathways, like RNA processing and the formation of RNA transport granules [28], and also in disease, like multisystem proteinopathy (MSP) and amyotrophic lateral sclerosis (ALS) [29]. The protein harbours a tandem pair of RRMs, whereas its highly repetitive LCD may also contain one or two RNA-binding motifs, as we came across in a sequence screen assessed by dedicated RBP predictors (G- and U-scale, NucBind) [30,31]. By direct binding analyses of these YGG-rich region(s), we show that they can bind a broad range of RNAs (both folded and unfolded) and can also bind DNA. Due to its binding, RNA has a strong influence on the LLPS of hnRNPA2 LCD. By a bioinformatics screen, we found similar regions in many other proteins classified as RNA- or DNA-binding proteins. The novel region we identified in hnRNPA2 is an imperfect repeat, rich in tyrosines and glycines. By determining the interaction strength of RNA with wild-type, all Tyr to Phe and all Tyr to Ala mutants of the imperfect YGG repeat, we show that aromatic residues are necessary for RNA binding, with F interacting even stronger than Y. Therefore, we confirm the YGG as a potential RNA-binding motif, and suggest to include phenylalanine in this model, by terming this nucleic-acid binding region ‘F/YGG motif’, and suggest that it may be a prevalent RBD in the RNA-binding complement of the proteome.

## Material and methods

### Constructs

hnRNPA2 LCD (R190 – Y341; UniProt P22626) (Addgene plasmid # 98,657; http://n2t.net/addgene:98657; RRID:Addgene_98,657), and hnRNPA2 LCD MBP(Addgene plasmid # 98,661; http://n2t.net/addgene:98661; RRID:Addgene_98,661) were a gift from Prof. Dr. N. Fawzy [32].

hnRNPA2 LCD D290V, hnRNPA2 LCD_∆NAID1_ and hnRNPA2 LCD_∆NAID2_ were cloned with Q5® site directed mutagenesis kit.

### Protein expression

All proteins were expressed in terrific broth with the appropriate antibiotic. BL21 STAR *E. coli* cultures containing the appropriate plasmid were grown at 37°C until OD 0.6–0.8. Afterwards, expression was induced with IPTG. hnRNPA2 LCD MBP bacterial pellets were harvested after 4 h of expression by centrifuging the cultures at 5000 x g for 20 minutes. For hnRNPA2 LCD and D290V, temperature was decreased to 25°C, and pellets were harvested after overnight expression.

### Protein purification

hnRNPA2 LCD was purified as described previously [33]. Briefly, after dissolving the pellet in lysis buffer (20 mM Tris-Cl, 500 mM NaCl, 10 mM imidazole, 1 mM dithiothreitol supplemented with 0.1 mM phenylmethylsulfonyl fluoride, 0.5 mM benzamidine hydrochloride, and 1 tablet Roche cOmplete EDTA-free protease inhibitor per 50 ml) and lysing through sonication, inclusion bodies containing hnRNPA2 LCD were dissolved in 3 M urea. The solution was cleared, filtered, loaded on 5 ml HisTrap column and eluted with a linear 0 mM to 250 mM imidazole gradient. The 6X HIS tag was cleaved of overnight by TEV protease. The solution was loaded again on 5 ml HisTrap column, and the flow through was run over gel filtration column (hiload 26/600 superdex 200pg). Pure protein was dialysed to 0.01 M CAPS pH 11.0 and flash frozen.

hnRNPA2 LCD D290V was purified similarly, with the exception that 8 M urea was applied, and the protein was desalted (HiPrep 26/10) to CAPS pH 11.0 buffer.

hnRNPA2 LCD MBP was purified as previously described [33]. Briefly, after dissolving the pellet in lysis buffer (20 mM HEPES, 500 mM NaCl, 10 mM imidazole, 1 mM dithiothreitol supplemented with 0.1 mM phenylmethylsulfonyl fluoride, 0.5 mM benzamidine hydrochloride and 1 tablet Roche cOmplete EDTA-free protease inhibitor per 50 ml). Bacterial debris was spun down, and protein was loaded onto 5 ml HisTrap column. hnRNPA2 LCD MBP was eluted with a linear 0 mM to 250 mM imidazole gradient, and protein was loaded onto gel filtration column (hiload 26/600 superdex 200pg). Pure protein was flash frozen and stored at −80°C.

### Peptides

Based on the YGG-rich region in hnRNPA2 LCD, we selected the second imperfect repeat region as a peptide model for F/YGG motif. Wild Type peptide (F/YGG WT) is the YGG model, F/YGG Y to F is the FGG model, and F/YGG Y to A serves as the negative control.

Peptides were purchased from Synpeptide (http://www.synpeptide.com/). Peptides were labelled with Nanotemper Monolith protein labelling Kit RED-NHS.

### RNA, DNA and RNase A

DNA (CAATAGTATGACAGTTCGAGG) was purchased from SigmaAldrich. To make it double stranded, its complement was added in equimolar concentration. The solution was heated to 99°C, and slowly cooled down.

PolyU (polyuridylic acid potassium salt) and polyA (polyadenylic acid) were purchased from sigma Aldrich. Yeast tRNA was purchased from Invitrogen. U30 was purchased from Eurofins. rRNA was purchased from bio-world.

### RNA purification

U2OS RNA was purified with TRIzole (Genebiotech). On a 10 cm dish with U2OS cells (>10^6^ cells), media was aspired and ice cold PBS was used to wash the cells. Next, 1 ml trizole was added and cells were scraped. The solution was transferred to an Eppendorf, and 250 µL chloroform was added. The solution was centrifuged for 10 minutes at 10,000 rpm. The clear, aqueous top layer – containing RNA – was carefully pipetted. The RNA was precipitated with isopropanol and washed with 70% ethanol. After drying the ethanol in a vacuum, the RNA was redissolved in DNase and RNase free water and stored at −80°C.

### RNA labelling

To fluorescently label RNA, the Pierce^TM^ RNA3’ End Biotinylation Kit (ThermoFisher Scientific) was used, but we replaced Biotinylated cytidine (bis)phosphate with cytidine-5’-phosphate-3’-(6-aminohexyl)phosphate, labelled with Cy5, Triethylammonium salt (Jena Bioscience).

DNase, and protease-free RNase A were purchased from ThermoFisher scientific.

### MST

Microscale thermophoresis (MST) measurements were performed on Monolith NT.115 using NT.115 Premium Coated capillaries. Assays were performed in 20 mM Hepes, 10 mM MgCl2, 0.05% Tween-20 pH 7.5. RNA (labelled U30) concentration was 420 nM. Protein concentration was titrated. MST experiments were run using the red laser at 50%. Initial laser off time was 5 seconds, followed by 30 seconds laser on time. The final laser off time was 10 seconds. Peptide experiments were performed with the same settings, but RNA was titrated against labelled peptide. MO Affinity Analysis was used to fit a Kd value. Experiments were performed in triplicates.

### Turbidity measurements

Except when clearly stated, all measurements were performed at a protein concentration of 20 µM. To induce LLPS, an appropriate volume of 0.5 M MES pH 5.5 was added to the protein solution. After a quick mixing, the turbidity of the solution was measured 600 nm (or 340 nm) on a BioTek Synergy^TM^ Mx plate reader at 25°C with continuous shaking over the course of 10 minutes. To give one value, the different turbidity measurements were averaged. Non-binding black 96 well plates of transparent bottom 540 (Greiner bio-one, chimney well, µclear®) were used, and covered with a transparent film (VIEWsealTM).

### DLS

Dynamic light scattering (DLS) measurements were carried out on a DynaPro NanoStar (Wyatt) instrument, together with a disposable cuvette (WYATT technology). Experiments were run at 25°C, for a prolonged period of time by collecting 10 acquisitions, 8 s each. DYNAMICS 7.1.9 was used to analyse the data. We used the regularization fit to get size estimates. During the analysis, the viscosity of the solution is assumed to be equal to that of water.

### Protein concentration

To determine the protein concentration, QUBIT^TM^ (ThermoFisher Scientific) was used. High-density phase was isolated by centrifuging the LLPS solution at 15.000xg for 5 minutes. Supernatant was carefully pipetted away, and the pellet was dissolved in 8 M urea.

### Fluorescent labelling of hnRNPA2 LCD

100 µl of 8 mg/ml protein solution was dialysed against 0.1 M sodium carbonate buffer, pH 8.5. 10 mg/ml of the fluorescent dye Dylight® 488 (Thermo scientific) dissolved in DMSO was added to the protein at a final concentration of 0.05 mg/ml and the solution was incubated at room temperature for 1 h. The solution was then dialysed against 0.01 M CAPS pH 11.0 storage buffer, to remove the excess of unbound fluorophore. Fluorescently labelled hnRNPA2 LCD was protected from light and stored at −80°C.

### Microscopy

Fluorescent microscopy measurements were carried out on a Leica DMi8 microscope equipped with a Leica 564 DFC7000 GT camera. Dylight® 488- labelled proteins were each mixed with 200x excess of the 565 same, non-labelled, protein. Phase separation was then induced by changing the pH of the protein solution as described earlier. The solution was incubated at 25°C and droplets were 567 visualized with 100x oil-immersion objectives with fluorescence microscopy (applying a FITC filter for protein, and Rho filter (red) for labelled RNA). ImageJ software was used to count droplets.

To perform fluorescence recovery after photobleaching (FRAP) experiments, stationary droplets were bleached (100% intensity, 500 ms bleach), and after bleach, a picture was taken every second. The fluorescence of single droplets was quantified with imageJ, and data was normalized using following formula:

$$recovery=\frac{I_{t}-I_{0}}{I_{A}-I_{0}}$$

With:

I_t_ = intensity at timepoint t

I_0_ = intensity at bleach

I_A_ = Intensity before bleach (= 100%)

### Lumicks C-trap

For fusion experiments, Lumicks C-trap device was used. Samples were injected in custom Lumicks microfluidic channels. Two 1064 nm optical trap lasers were used at 10% power each to trap 2 droplets, and move them against each to force fusion. Images were acquired using a laser scanning confocal microscope, with avalanche photodiode fluorescence detectors with single-photon sensitivity, using a laser with 488 nm excitation power.

### Denaturing PAGE

PolyU RNA (with or without hnRNPA2 and/or RNase A) was diluted in RNA gel loading dye (ThermoFisher Scientific) and boiled. Afterwards, they were run on 10% TBE polyacrylamide gels supplemented with 8 M urea at 150 V. RNA was stained with SYBR gold (ThermoFisher Scientific).

### Bioinformatics analysis

Primary protein sequence was scored with the G- and U-scale [30] using the VOLPES webserver (https://homepage.univie.ac.at/lukas.bartonek/testserver/app.html) [34]. Scores were averaged with a running window of 21 amino acids (default). NucBind [31] was also used to predict putative DNA- and RNA-binding regions in the sequence. Both G-scale and NucBind predicted the C-terminal half of the protein as nucleic acid-interacting region. These prediction results are in consensus with those of RNAbindRplus [35] and DP-Bind [36].

Intrinsically disordered protein regions were predicted by IUPred2 [37] using the default ‘long disorder’ setting. The C-terminal half of the protein was predicted to be disordered. This prediction for the hnRNPA2-LCD is in perfect alignment with the output of other disorder predictors, namely ESpritz-NMR [38], JRONN [39] and VSL2b [40].

Posttranslational modifications of hnRNPA2ʹs LCD were retrieved from PhosphoSitePlus v6.5.9.3 [41]. No downstream filtering was made in addition.

A poly-phosphotyrosine mimetic construct (phospho-mutant) was generated by replacing the annotated (PhosphoSitePlus) phosphotyrosines of the LCD with aspartates.

UniProt’s BLAST was run against all human proteins in SwissProt searching for homologous sequences with the hnRNPA2ʹs repeat:

GYGSGRGFGDGYNGYGGGPGGGNFGGSPGYGGGGPGYGNQGGGYGGGYDNYGGGNYGSG

E-value threshold was set to 0.01 and gapped alignment was allowed.

In total, 136 sequences were found including hnRNPA2. The hits included 37 keratin and keratin-associated proteins that were excluded from the subsequent analysis. The rest of the proteins were analysed in terms of functional enrichment. Panther Protein Class enrichment analysis against the human proteome was carried out using Fisher’s exact test with Bonferroni correction for multiple testing.

The imperfect repeat was identified by RADAR [42]. To identify regions in FUS and hnRNPA1 which are similar to the imperfect repeat, all regions with an alignment score above 45 were pooled.

Protein BLAST was used to align the imperfect repeat from hnRNPA2 homologues.

### TEM

Transmission electron microscopy was performed to track the morphological properties of hnRNPA2 LCD D290V. Solutions of (10 μl) protein were adsorbed for 15 min to formvar film coated 400-mesh copper grids (Agar Scientific Ltd., England), following a short glow discharge step to improve adsorption. Grids were subsequently washed with of milli-Q water and next stained with uranyl acetate (2% w/v) for 1 minute. Excess stain was removed by blotting with a filter paper and samples were left to air dry. The grids were examined using a JEM-1400 120 kV transmission electron microscope (JEOL, Japan) operated at 80 keV.

### Data visualization and analysis

All graphs were created and analysed using GraphPad Prism 8.

## Results

### hnRNPA2 LCD interacts with RNA

**Figure 1.**
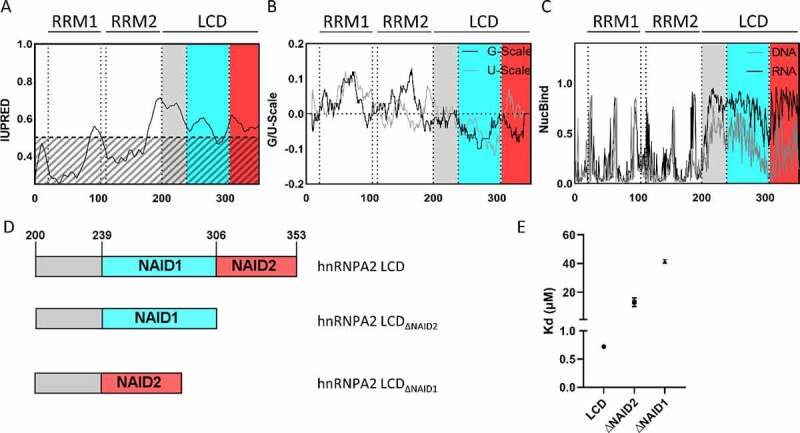
hnRNPA2 LCD interacts with RNA.

IDPs/IDRs lack a single, well-defined tertiary structure and can assume an ensemble of highly dynamic conformational states, a feature that can be successfully predicted from amino acid sequence. By a dedicated ID predictor of structural disorder (IUPred2) [37], hnRNPA2 clearly has two N-terminal folded domains (RRMs), and an IDR in its C-terminal half (Fig. 1A). By another sequence-based predictor, SEG [43], the disordered region is also of low sequence complexity (i.e. satisfies the criterion for a low-complexity domain, LCD) [44]. The structural disorder of the region has already been studied experimentally by NMR spectroscopy [32], and the unstructured nature of the region was confirmed.

**(A)** IUPred2 confirms structural disorder of the C-terminal LCD of hnRNPA2, **(B)** the G-scale and to a lesser extent the U-scale predict two potential RNA interaction regions (we termed NAID1 (239–306) and NAID2 (307–353) marked by colour). **(C)** This prediction was confirmed by NucBind-DNA and -RNA.**(D)** Three constructs were generated to test which of the two predicted RNA domains interacts with RNA *in vitro*: full-length LCD, hnRNPA2 LCDΔNAID1 (region 239–306 deleted) and hnRNPA2 LCDΔNAID2 (region 307–353 deleted). **(E)** Dissociation constants of hnRNPA2 LCD, and the two deletion constructs for U30 RNA, were measured by MST (cf. Supplementary Fig. S1), Kd_LCD_ = 0,72 ± 0,002 µM; KdΔ_NAID2_ = 13 ± 3,0 µM; KdΔ_NAID1_ = 41 + 1,4 µM. Error bars represent standard deviation of triplicate experiments.

We have screened the hnRNPA2 sequence with complementary sequence-based predictors, G- and U-scales, especially developed to score amino acid–RNA interaction preferences. These scales are based on known structures of RNA/protein complexes, and provide a statistical analogue of relative binding free energy, with negative values corresponding to higher affinities [30]. For example, it can predict known disordered RNA-binding domains in FUS [45]. By applying these scales on hnRNPA2 sequence, we observed two potential RNA-interacting regions within its LCD (200–353), namely from residue 239–306 and from 307–353 (Fig. 1B). This result was strengthened by two variants of another predictor, NucBind-RNA and NucBind-DNA (Figure 1C) [31,34]. Because of this prediction, and also because LCDs are enriched in RNA- and DNA-binding proteins [14], we did not rule out DNA binding, and therefore termed the two regions (putatively) Nucleic-Acid Interacting Domains (NAIDs). Interestingly, within these domains, there is an enrichment of YGG motifs.

To validate these predictions, we used microscale thermophoresis (MST) to demonstrate RNA binding by the LCD and to assess the contribution of NAIDs (by U_30_ as an RNA model). To this end, we compared the dissociation constants (Kd) of full-length LCD, a construct in which the first putative NAID was deleted (hnRNPA2 LCD_∆NAID1_), and a construct in which the second putative NAID was deleted (hnRNPA2 LCD_∆NAID2_) (Fig. 1D). We found that hnRNPA2 LCD binds U_30_ with a low-micromolar affinity (0.72 µM), whereas binding of hnRNPA2 LCD_∆NAID1_ is much weaker, with a high-micromolar affinity for RNA (41 µM) (Fig. 1E). Deletion of NAID2 has much less effect on the RNA binding of LCD, as the Kd of hnRNPA2 LCD_∆NAID2_ is comparable to that of the wild type (13 µM) (Fig. 1E, Supplementary Fig. S1), confirming the importance of NAID1 in RNA binding. Interestingly, NAID1 contains only 1 RGG motif, whereas 3 out of 4 RGG motifs in the LCD are not located within the NAIDs, suggesting that other motifs might be involved in the interaction.

### Effect of RNA on the LLPS of hnRNPA2 LCD

**Figure 2.**
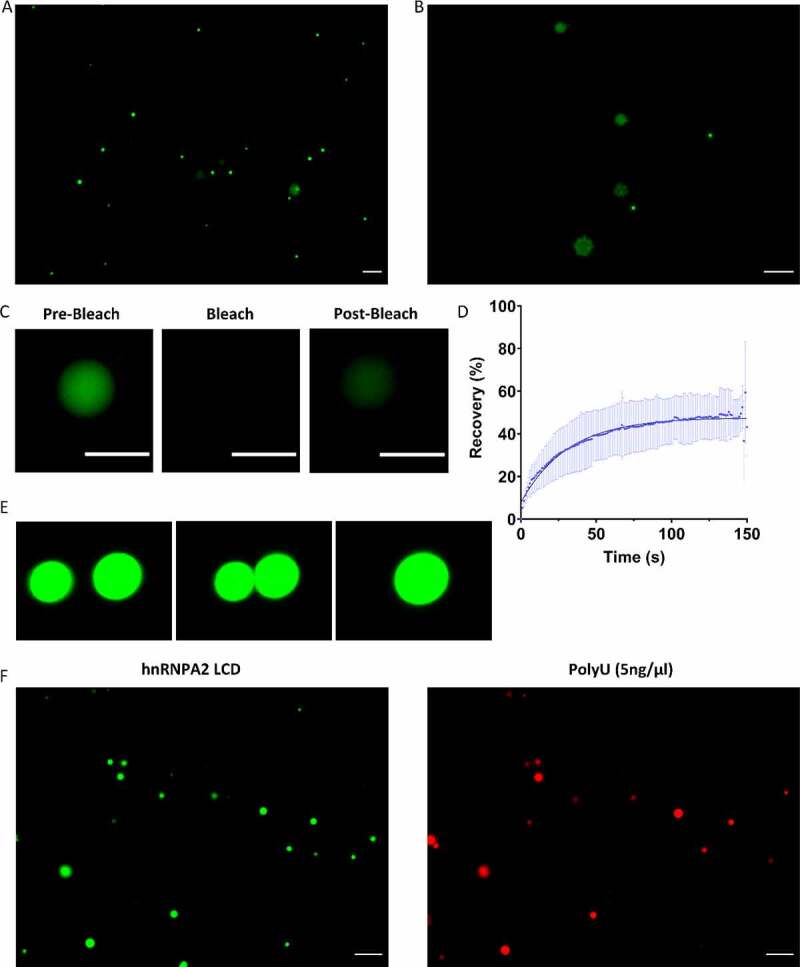
hnRNPA2 LCD undergoes LLPS and recruits RNA to droplets.

hnRNPA2 is implicated in LLPS leading to the formation of liquid-like organelles including mRNA transport granules, the nucleolus and stress granules [46–48]. Within these, it can act both as a scaffold and a client protein [49]. At physiological pH, protein-rich droplets quickly appear in the solution of hnRNPA2 LCD, showing that hnRNPA2 LCD is prone to undergo phase separation (Fig. 2A). Over time, these droplets mature and turn into aggregates (Fig. 2B). One hour after inducing phase separation, the droplets show 50% recovery of their fluorescence in fluorescence recovery after photobleaching (FRAP) experiment, illustrating the liquid-like nature of droplets (Fig. 2C, D). The liquid-like nature is also confirmed by showing effective fusion of two adjacent droplets by C-trap experiments (Fig. 2E). When adding polyU RNA to hnRNPA2 LCD droplets (LCD binds a broad range of RNAs, see later), they co-phase separate, showing that hnRNPA2 LCD not only binds RNA, but also recruits the RNA into phase-separated droplets (Fig. 2F).

**(A)** After bringing the pH of a 20 μM hnRNPA2 LCD (green) solution from 11 to 7, protein-rich droplets form, which **(B)** turn over time into aggregates. **(C)(D)** Protein-rich droplets show an about 50% recovery of their fluorescence in FRAP, confirming their liquid-like nature. **(E)** Optical tweezer-controlled fusion of two droplets. **(F)** After inducing LLPS, the hnRNPA2 LCD (green) droplets are able to recruit polyU RNA (red) at a low concentration. Scale bar represents 10 μm. Error bars represent standard deviation of four replicates.

**Figure 3.**
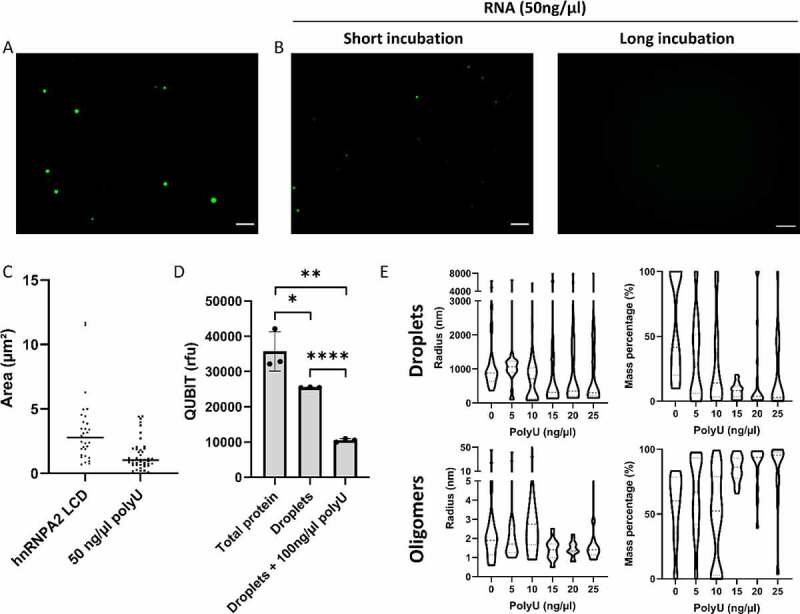
RNA increases the saturation concentration of the LLPS of hnRNPA2 LCD.

RNA promotes LLPS in the case of many phase-separating proteins, but often its effect goes through an optimum (showing ‘reentrant’ behaviour), mostly attributed to charge screening at high RNA:protein ratios [50–52]. In accord, when we add RNA at a higher concentration, droplet size decreased significantly and aggregates are not observed over time in any of the samples (Fig. 3A, B, C).

**(A)**One hour after inducing LLPS of 20 µM hnRNPA2 LCD, big protein-rich droplets form. **(B)** The addition of polyU at a high concentration (50 ng/ml) decreases droplet size, and after longer incubation, aggregation was not observed (scale bar represents 10 µm). **(C)** The decrease in droplet size is statistically significant (Mann–Whitney test, *p* ≤ 0.05). **(D)** QUBIT® showed a significant decrease in the protein concentration of the high-density phase upon adding 100 ng/ml polyU, showing that polyU significantly increases hnRNPA2 LCD saturation concentration. Unpaired t-tests were performed to show significant differences (*p ≤ 0.05; **p ≤ 0.001; **** *p* ≤ 0.0001) **(E)** Dynamic Light Scattering (DLS) was used to determine droplet size. Everything above 50 nm in diameter was considered a droplet, whereas every object below 50 nm was termed an oligomer. Increasing polyU concentration has led to a decrease in droplet radius, and the total amount of protein in droplets. Increasing polyU concentration had no influence on monomer size, but increased the amount of oligomer.

These results show that RNA at high concentrations directly affects droplet size, whereas it cannot be ascertained if the total protein concentration in droplets remains unchanged, or RNA increases the dilute phase concentration (or concentration of protein dissolved in solution) leading to a decrease in protein concentration in high-density droplets. To investigate this question, we designed directed experiments to determine which hypothesis holds true.

By sedimenting high-density droplets and determining their protein concentration, we could show that the total amount of hnRNPA2 LCD decreases in the high-density phase, i.e. less hnRNPA2 LCD undergoes LLPS and RNA indeed increases the dilute phase concentration (Fig. 3D). We confirmed this conclusion by dynamic light scattering (DLS), where we saw a decrease in droplet size and total protein concentration in droplets upon increasing RNA concentration (Fig. 3E). Interestingly, not all droplets dissolved, rather some droplets with a radius around 300 nm persisted, suggesting some droplets (or compartments within the droplet) are less sensitive to RNA. In keeping with this interpretation, the number of hnRNPA2 LCD oligomers (objects smaller than 50 nm) and monomers (objects of around 2 nm) increased [53]. As expected, RNA had no effect on the size of these oligomers, which provides additional proof that RNA increases the dilute phase concentration of hnRNPA2 LCD (Fig. 3E).

### hnRNPA2 LCD interacts with different nucleic acids

**Figure 4.**
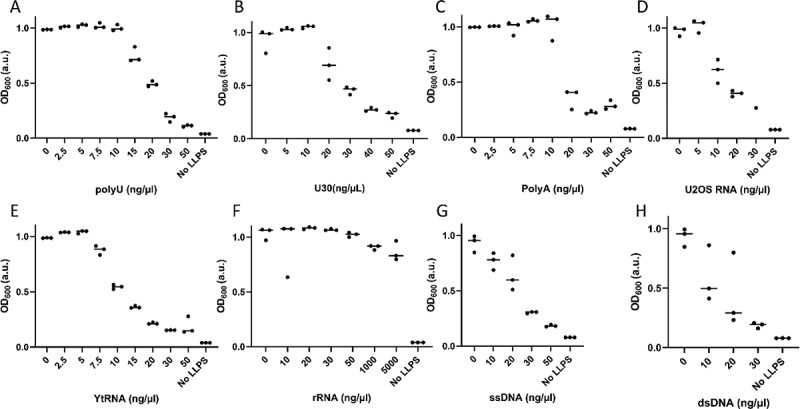
hnRNPA2 LCD interacts with many types of RNA and also DNA.

To test whether the increase of the dilute phase concentration of hnRNPA2 LCD LLPS is specific to polyU, we quantified the effect of a range of RNA variants on LLPS by measuring the turbidity (absorbance at 600 nm, OD600) of the solution. Without RNA, OD600 shows a high value (around 1.0), characteristic of the formation of small droplets. Upon increasing RNA concentration, the turbidity starts to decrease at a particular concentration where droplets disappear (Fig. 4A) (cf. Fig, 3A, B). The effect of RNA does depend slightly on its size, as short polyU repeats of 30 nucleotides (U30) have a similar effect (Fig. 4B), although at a slightly higher concentration, suggesting that larger RNA molecules are more efficient in increasing the dilute phase concentration of LLPS. PolyA is somewhat more effective than polyU (Fig. 4C), showing that the interaction is not nucleotide-specific. Total purified RNA from a human cell line (U2OS) is also very effective (Fig. 4D). Motivated by observations that suggested the preference of LLPS for disordered RNA [54], we also tested structured (folded) tRNA, which actually seemed to be more effective at increasing the dilute phase concentration (Fig. 4E). Interestingly, highly structured rRNA has no effect on LLPS, even at high concentrations (Fig. 4F). On the other hand, both single-stranded and double-stranded DNA influence LLPS at least as effectively as RNA (Fig. 4G, H), suggesting that NAIDs constitute a general nucleic acid – rather than strictly RNA – binding motif. In summary, all studied nucleic acids influenced LLPS in a similar manner, except for rRNA, which appears to have no effect, apparently because it does not bind to LCD.

We used turbidity readout (OD600) as a measure of the effect of nucleic acids on the LLPS of hnRNPA2 LCD. We showed above RNA increases the protein saturation concentration. This leads to a decrease in OD600 value, therefore indicating a decrease in LLPS. We measured the effect of increasing concentrations of: **(A)** polyU, **(B)** Repeat uracil RNA of a length of 30 nucleotides (U30), **(C)** polyA, **(D)** whole-cell (U2OS) **(E)** transfer RNA (tRNA). **(F)** Ribosomal RNA (rRNA) did not have an influence on LLPS, even at high concentrations. **(G)** Single-stranded and **(H)** double-stranded DNA affected LLPS to an extent comparable to RNA.

Interestingly, at very low hnRNPA2 LCD concentrations RNA promotes LLPS, showing that RNA decreases the saturation concentration (Supplementary Fig. S2). Probably at such low protein concentration, RNA allows for multivalent interactions between different protein monomers, whereas it would inhibit protein–protein interactions at higher concentrations. It is, however, worth noting that, because of high intracellular hnRNPA2 concentration, this only happens well below physiological hnRNPA2 levels.

### Electrostatic and hydrophobic components of the interaction between hnRNPA2 LCD and RNA

**Figure 5.**
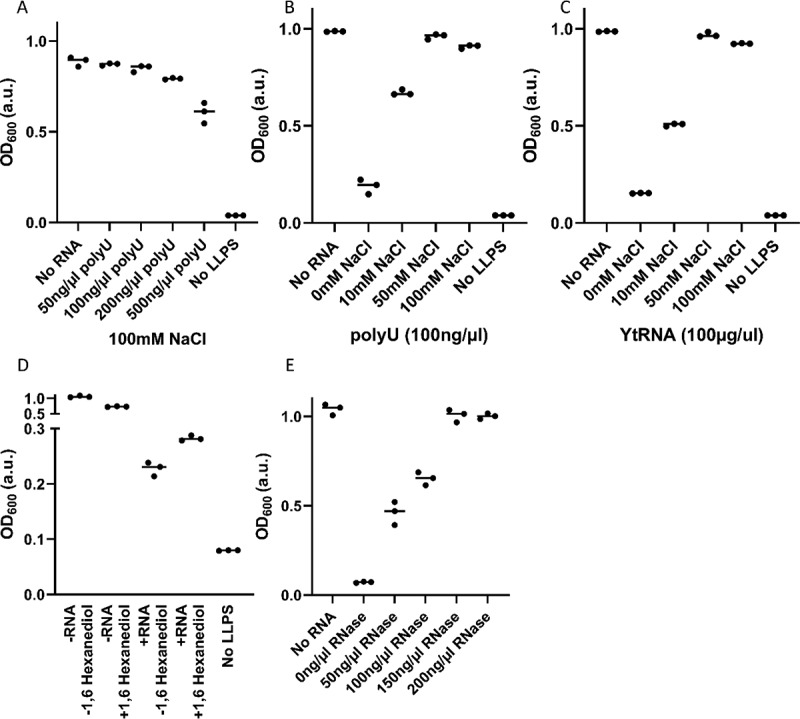
Salt, 1,6 hexanediol and RNAse A reverse the LLPS-inhibitory effect of RNA.

While the interaction exists at physiological salt concentrations (Fig. 5A), to study the nature of hnRNPA2 LCD:RNA interaction, we change protein:RNA ratio not to saturate the protein with RNA, and investigated if salt and 1,6 hexanediol interfere with the effect of polyU and tRNA on the LLPS of hnRNPA2 LCD. We found that NaCl inhibits the effect of both folded and unfolded RNA (i.e. it recovers LLPS) (Fig. 5B, C). Under the given conditions, low ionic concentrations already have a major influence on the interaction, which probably means that only a few key residues need ionic coverage for the interaction to be inhibited. 1,6 hexanediol is an aliphatic alcohol, which can compete with weak hydrophobic interactions, inhibiting the LLPS of various proteins, such as TDP-43 [55], FUS [56] and huntingtin exon1 [57]. Here, we show it has a direct effect on the LLPS of hnRNPA2 (decreasing its OD600 from about 1.0 to 0.7), whereas it can also counteract the LLPS-inhibitory effect of RNA, causing a small increase in turbidity (Fig. 5D). Thus, hydrophobic interactions do contribute to the interaction of hnRNPA2 LCD and RNA. To demonstrate that the observed effects are due to an interference with protein–RNA interaction, we show that degradation of the nucleic acid completely reverses RNA effect, recovering LLPS of LCD with increasing RNase A concentration (Fig. 5E, and Supplementary Fig. S3).

**(A)** polyU interferes with 20 µM hnRNA2 LCD LLPS at a physiological NaCl concentration (100 mM). **(B)** NaCl inhibits the effect of both polyU RNA **(C)** and tRNA on the LLPS of hnRNPA2 in a concentration-dependent manner. **(D)** 1,6-hexanediol slightly decreases the LLPS of hnRNPA2 LCD, but increases residual LLPS in the presence of polyU (100 ng/ml), i.e. it reduces LCD:RNA binding. **(E)** RNase A completely reverses the effect of RNA.

### Effect of RNA on the LLPS of hnRNPA2 LCD carrying the disease-linked D290V mutation

**Figure 6.**
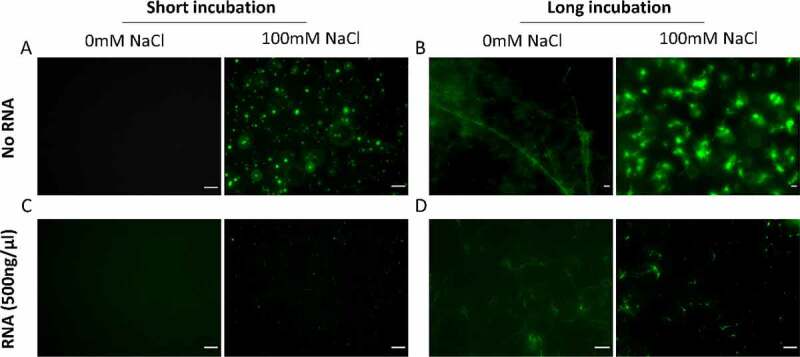
The effect of RNA on the LLPS of ALS-related D290V mutant of hnRNPA2 LCD.

Next, we addressed if the observed effect of RNA also applies to the LCD carrying a disease-associated mutation, D290V. This mutation has been suggested to affect LLPS and to promote the aggregation tendency of LCD [58]. It is of special interest with regard to RNA binding, because this mutation falls within NAID1. We observe that without salt, hnRNPA2 LCD D290V does not phase separate at pH 7.0, rather it forms fibrous aggregates within a few hours, whereas at physiologically relevant NaCl concentrations (100 mM), it potently phase separates and forms amorphous aggregates (Fig. 6A, B; Supplementary Fig. S4). As expected, RNA has no effect on D290V in conditions that did not allow LLPS. However, similarly to wild-type LCD, RNA decreases phase separation under conditions that favour LLPS (Fig. 6C, Supplementary Fig. S5). In contrast to wild-type LCD, some aggregation is still observed (Fig. 6D), although aggregates are smaller and less abundant. In conclusion, RNA does interact with LCD D290V and limits its aggregation, but it cannot completely stop it.

The effect of RNA on LLPS is comparable to that observed with wild-type LCD. **(A)** At pH 7.0, hnRNA2 LCD D290V requires physiologically relevant NaCl concentrations (100 mM) to undergo LLPS. **(B)** Overnight, aggregation is observed in both low-salt and high-salt conditions. Aggregates in the two conditions do differ, those formed in the presence of LLPS have a more amorphous morphology, while those formed without LLPS look fibrous. **(C)** polyU (500 ng/µl) decreases the size and number of droplets and **(D)** results in much smaller aggregates overnight. Scale bar represents 10 µm.

### F/YGG motif is a low complexity nucleic-acid interaction domain

**Figure 7.**
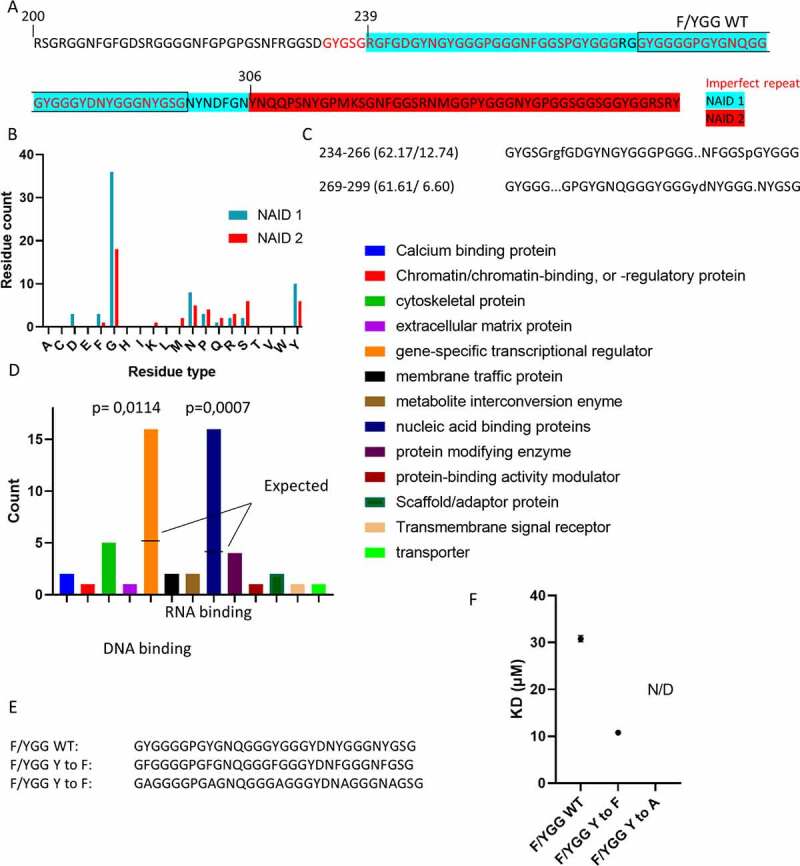
Conservation of YGG motif in human proteins.

Our results show that the middle section (region 239–306 termed NAID1) is a distinguished nucleic acid-binding region of the LCD of hnRNPA2 being the main driver of LCD binding a broad range of RNA and DNA molecules (Fig. 7A). NAID1 contains an increased amount of Tyr residues adjacent to Gly residues (Fig. 7B), which is much less present in NAID2, suggesting that these YGG patches may play an important role in nucleic acid binding. Furthermore, we observed that NAID1 is actually constituted of two tandem copies of an imperfect repeat sequence of about 30 residues, rich in YGG motifs (Fig. 7C). This region is highly conserved in hnRNPA2 homologues (Supplementary Fig. S6A), suggesting that it is the elementary nucleic acid binding region within hnRNPA2 LCD. We suggest to term this region, in reflection of its peculiar repetitive amino acid sequence, the F/YGG motif. To provide direct evidence for this tenet, we tested RNA binding by this region.

**(A)** The sequence of hnRNPA2 LCD (res. 200–353 of full-length hnRNPA2, cf. Fig. 1A), with two potential nucleic acid interacting domains NAID1 and NAID2 highlighted, and **(B)** their amino acid composition compared. **(C)** NAID1 constitutes two tandem copies of an imperfect repeat rich in GY and YGG patches, which we suggest to term ‘F/YGG motifs’.**(D)** By a BLAST search for regions homologous to the consensus sequence of YGG repeat in the human proteome, we found that it is present in many other, primarily RNA- and DNA-binding, proteins. On panel D, proteins mapping to ‘nucleic acid binding proteins’ were all mapped to the subcategory ‘RNA-binding proteins’ as well, while proteins mapping to ‘gene-specific transcriptional regulator’ were mapped to the subcategory ‘DNA-binding transcription factors’ with one single exception. **(E)** Sequence of the wild-type second imperfect repeat (F/YGG WT), or with Y replaced by F (F/YGG Y to F), or by A (F/YGG Y to A). **(F)** Dissociation constants between RNA and F/YGG WT, F/YGG Y to F and F/YGG Y to A were measured by MST (cf. Supplementary Fig. S7). KD_F/YGG WT_ = 31 ± 0,7 µM; KD_F/YGG Y to F_ = 11 ± 0,5 µM; KD_F/YGG Y to A_ = N/D (not determinable; while binding could be detected, it was too weak to measure).

As a F/YGG model, we chose the second repeat (residues 269–299). We call this region F/YGG WT. By MST (Supplementary Fig. S7), the F/YGG motif interacts with RNA with a Kd of 31 μM. As hydrophobic interactions are important in NAID1 – RNA interaction (Fig. 5D), we next tested direct RNA interaction of the F/YGG motif in which all tyrosine residues were mutated to either phenylalanines (F/YGG Y to F) or alanines (F/YGG Y to A, cf. Fig. 7E). The motif with Y to F mutations binds RNA even stronger than wild type (Kd = 10 μM), whereas all Ala mutation of Tyr-s practically abrogates RNA binding (Fig. 7F).

Next, we performed a BLAST search among Swiss-Prot human proteins to determine if this imperfect repeat region (F/YGG motif) was also present in other proteins. With hnRNPA2 included, we got 137 hits (100 without keratin-like and keratin-associated proteins, for details see the Methods), among which RNA- and DNA-binding proteins were highly significant (Fisher’s exact test Bonferroni corrected p-values: p = 0.0114 and p = 0.0007) (Fig. 7D) enriched. hnRNPA1, for the LCD of which nucleic acid binding was already suggested [25–27], and FUS score high on this list, and as expected these homologous proteins show very high GY content, and to a lesser extent enrichment in Phe, Ser, Asn and Gln residues (Supplementary Fig. 6B,C). This result creates a strong link between nucleic acid binding and the presence of F/YGG motifs, which suggests targeted studies of this feature in the hnRNP family (Fig. 7D).

## Discussion

In this study, we report that hRNPA2 LCD interacts with a wide range of RNA and DNA molecules, via a conserved region located in the middle of the LCD. Interestingly, this region carries a few Arg residues often observed in disordered RNA-binding proteins [59], but here its defining feature rather appears to be an imperfect repeat of about 30 residues with a high abundance of Tyr-Gly and Gly-Tyr patches. We expect these regions to mediate RNA binding, because Gly, having an exposed backbone, can engage in a significant amount of contact with nucleic acids [60], whereas tyrosines are also involved in DNA interactions [61]. The YGG motif has been proposed as a potential RNA-binding motif that contributes to RNA chaperone activity. Here, we demonstrate and suggest that these YGG patches (F/YGG motifs) are of key significance for LCD-nucleic acid interactions. Other amino acids observed in this region are Ser, Asn and Gln, all of which are prone to engage in hydrogen bonding with nucleic acids [60].

Because YGG patches have also been suggested to drive protein–protein interactions, a YGG region can support multivalent inter-protein interactions – leading to LLPS [62]. Nucleic acids have a profound inhibitory effect on this type of protein–protein phase separation as they can outcompete the protein–protein interaction. We have found similar motifs in many RNA- and DNA-binding proteins (including FUS, and hnRNPA1). Alternative to F/YGG-rich patches, strict FG-patches could also potentially serve the same function as F/YGG, for example, there are six FGs present in TDP-43 LCD [52], and the FG repeat in FG-NUPS, as we prove the importance of both tyrosine and phenylalanine for RNA interaction in this region. Therefore, we state that the F/YGG motif serves as a novel, nucleic-acid interaction domain, which could also cooperate with other protein domains (e.g. nearby RRMs) in DNA and RNA binding. Our observations show that LLPS is highly sensitive to RNA, thus it appears that the F/YGG motifs may allow to create beneficial, highly tunable LLPS regulatory circuits. Because the effect of nucleic acids on LCD LLPS is very sensitive to salt and 1,6-hexanediol, we hypothesize that the interaction is mediated via a few key residues only.

Interestingly, the F/YGG motif is related in primary sequence to the [G/S]Y[G/S] domain and, as stated above, to the FG domain. The [G/S]Y[G/S] sequence element has been identified as important for LLPS and aggregation. The Tyr can be changed to Phe, highlighting the importance for aromatic stacking. In the case of the F/YGG motif however, it is one (or more) glycine(s) neighbouring the aromatic residue, never a serine. However, while there can be an easy evolutionary switch from glycine to serine (only one nucleotide change), this switch is not observed even in distant hnRNPA2 homologues. Thus, while the modes of action of these three motifs are probably very related, they are not interchangeable, but three distinct motifs [63,64].

It is also important to note the role of phosphorylation as a key mechanism of the aforementioned regulatory circuits. How phosphorylation suppresses the LLPS of the hnRNP protein family is a highly studied question in the field [65–67]. Interestingly, threonines are fully depleted in the LCD of hnRNPA2, thus the 28 annotated phosphorylations in PhosphoSitePlus (v6.5.9.3) [41] is divided between phosphotyrosines and phosphoserines (14 + 14). This means that 28 out of the 33 Ser/Tyr residues are phosphorylated (85%). The prevalence of glycines flanking the phosphosites (GpY, dYG, GpS, pSG) is also striking, especially for phosphotyrosines: 11/14 pY sites are found to be flanked by glycines, while phosphoserines are also often adjoined glycines (10/14) (Supplementary Fig. S8A). Supposably, glycines help increase the accessibility of these phosphosites for kinases. Based on these observed trends, it is tempting to hypothesize the model that multisite phosphorylation acts as a switch between the more LLPS-prone and the less LLPS-prone form of the LCDs of hnRNPs, by decreasing the RNA-interaction affinity. This assumption is to be confirmed experimentally, but our bioinformatics predictions (NucBind, U-scale) suggest a significant decrease in the RNA-binding propensity for the construct with phosphomimetic pY~E phosphosites (p = 0.0156 & p = 3*10^−33^, respectively, one-tailed paired t-test) (Supplementary Fig. S8B).

By addressing the effect of various nucleic acids on the LLPS hnRNPA2 LCD, we found that all types of RNA, both folded and unfolded, and independent of sequence, had a similar influence, namely increasing the dilute phase concentration, and decreasing the number and size of droplets. The only exception is rRNA, which had no influence on LLPS. Of possible relevance, it has been seen before that as riboparticles mature towards rRNA, they become less phase separation prone [51], and rRNA is also unable to induce LLPS of G3BP1, which is an important driver for stress granule formation [54]. Thus, it could be a general trend that mature rRNA behave different from other RNA species and are less prone to be involved in LLPS probably due to its highly organized structure that shields nucleic acid bases from interaction with Tyr and Phe residues of potential partner proteins. The increase of dilute phase concentration was also observed in the case of single-stranded and double-stranded DNA. hnRNPA2 has been shown in the past to interact with ssDNA [68], and our results suggest that its LCD may have an important role in this interaction.

In general, RNA has been observed to play crucial roles in both promoting and inhibiting LLPS in a concentration-dependent manner, with higher RNA concentrations generally inhibiting LLPS [50–52]. Furthermore, RNA-dependent suppression of LLPS has been observed in several hnRNPs, such as FUS, TIA-1, TDP-43 and hnRNPA1 [69]. These proteins all contain RRMs, and studying the full-length proteins made it difficult to dissect and appreciate the possible contribution of their LCD to RNA (nucleic-acid) binding. Here, for the first time, we could show that the LCD of hnRNPA2 itself can encode for RNA binding and RNA buffering capacity.

As about half of RNA-binding proteins have no identified RNA-binding domains, and much of such regions fall into disordered regions, the F/YGG repeat motif may be an important addition to the growing list of RNA-binding motifs [2]. Further, numerous RBDs, such as those with Zn-finger domains [2], bind both RNA and DNA, and the F/YGG repeat motif of hnRNPA2 LCD also has this double capacity, and its potential general importance is underscored by that it is enriched in a broad range of RNA- and DNA-binding proteins, i.e. it can also be an important RNA chaperone [16], DNA-binding element for transcription factors, and other DNA-interacting proteins. Noteworthy, LLPS of transcription factors also often happens in so-called super-enhancers [70], and Y- and F-rich repeat regions could help transcription factor(s) tether to the DNA, decreasing the distance between the specific DNA-interacting domains and the DNA.

An interesting aspect of this RNA-binding IDR domain may derive from the fact that hnRNPA2 also contains other, canonical RNA-binding domains, RRMs. As affinity and specificity of a single RBDs is often not sufficient to provide selective binding *in vivo*, RBPs typically have modular architecture containing multiple RNA-interacting regions [13], enabling cooperativity and specificity in binding [2]. A further interesting aspect of intrinsically disordered RBPs is that they also occur multiple times (repeated internally), further increasing the resulting coordination and cooperativity [14]; this is exactly what we observe in the case of F/YGG motif in hnRNPA2 LCD.

With regard to mechanistic details of the interaction of F/YGG motif and RNA, protein–RNA interactions most often rely on electrostatic attraction and hydrogen bonds, whereas hydrophobic interactions between RNA bases and hydrophobic side provide further stabilization. In certain cases, hydrophobic interactions may account for 50% of the protein–RNA interface, in the form of pi-pi stacking [2]. It has been speculated that promiscuous proteinRNA binding may be mediated by electrostatic interactions, whereas sequence-specificity may be built in interactions with the nucleotide bases and shape complementarity [15]. As we provide evidence for the direct involvement of aromatic residues (Y and F) in the F/YGG motif, we suggest its inherent specificity, which may explain its widespread phylogenetic occurrence. The observed sensitivity of the effect of RNA on the LLPS of hnRNPA2 LCD to ionic strength and 1,6-hexanediol suggests the contribution of both factors here.

It is to be noted that the excessive presence of Gly and aromatic residues could also have a dangerous edge. Namely, it has been shown that Gly is intrinsically destabilizing in beta sheets. However, pairing of Gly with Phe or Tyr allows the presence of Gly in beta sheets to be thermodynamically favourable. Therefore, the excessive presence of Gly + aromatic residues could lead to beta sheet aggregation. Indeed, LARK domains (Low-complexity Aromatic-Rich Kinked Segments) are highly aggregation prone, and enriched in similar residues [71]. Conclusively, while they have an obvious beneficial function in the protein, these domains also contribute to the high aggregation potential of these domains. The evolutionary prevalence of F/YGG motifs in RBPs and DNA-binding proteins, however, shows this danger is mitigated by nature, and F/YGG motifs expand the list of RBDs as novel, general nucleic acid binding motif.

Its occurrence in a broad range of proteins raises interesting structural, molecular and functional question we plan to scrutinize in the near future. To conclude, we show the F/YGG motif as a novel, important disordered DNA- and RNA-interaction domain, as exemplified by hnRNPA2 LCD.

## Supplementary Material

Supplemental Material

## Data Availability

The authors confirm that the processed data supporting the findings of this study are available within the article or its supplementary materials. The raw data of this study are available from the corresponding author, JVL, upon reasonable request.
