# Supplementary material for: F/YGG-motif is an intrinsically disordered nucleic-acid binding motif
Source: RNA Biol. 2022 May 1;19(1):622–35. doi: 10.1080/15476286.2022.2066336 (PMC9067507; doi:10.1080/15476286.2022.2066336)
Supplement: Supplemental Material [file KRNB_A_2066336_SM7222.docx]

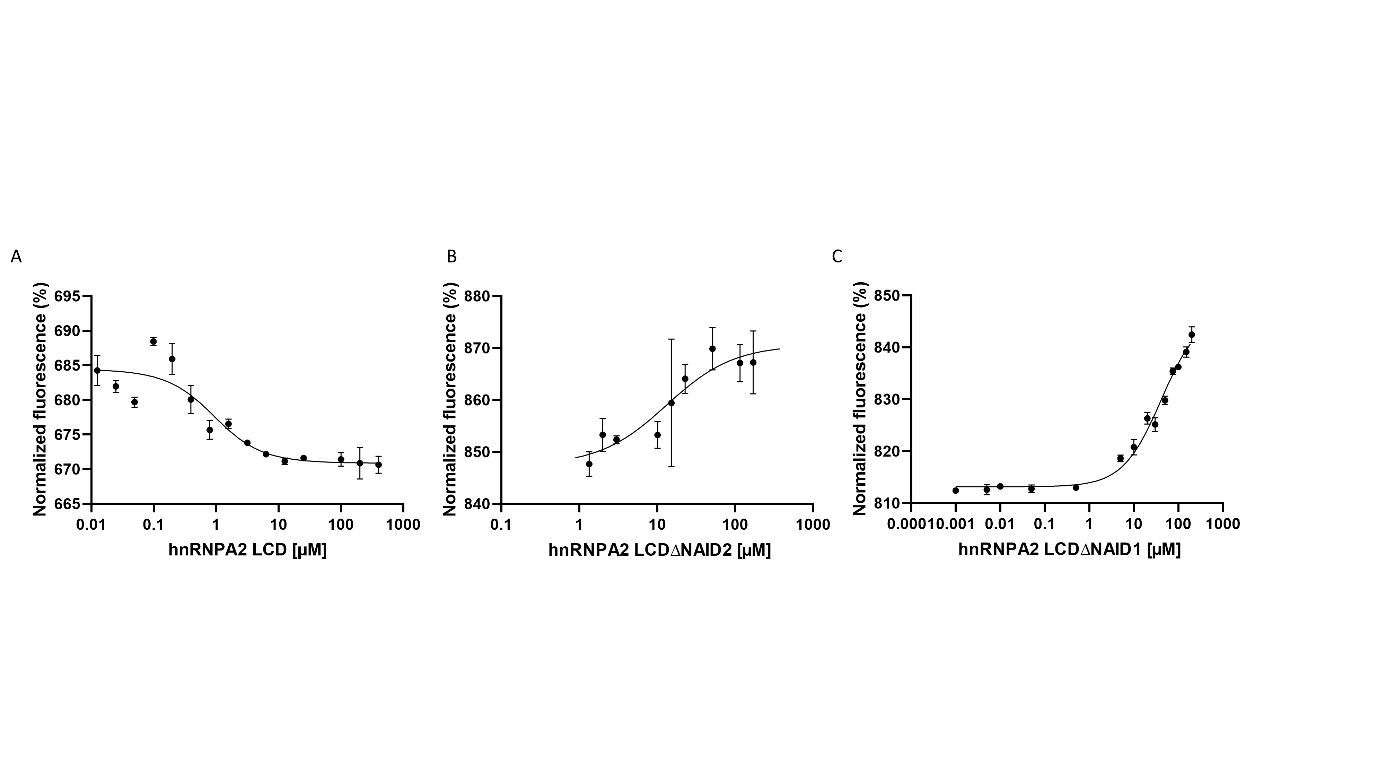


**Supplementary Figure 1**

MST data of hnRNPA2 LCD, hnRNPA2 LCDΔNAID2 and hnRNPA2 LCDΔNAID1 , used to calculate the dissociation constants represented in figure 1. Error Bars represent standard deviation from triplicate experiments.

**Supplementary Figure 2**
Turbidity (OD340) as a readout of the effect of RNA on hnRNPA2 LCD LLPS at low protein concentration. Comparable to figure 6, RNA increased the LLPS of hnRNPA2 LCD only at 0.5µM. PolyU at 20ng/µL was used.

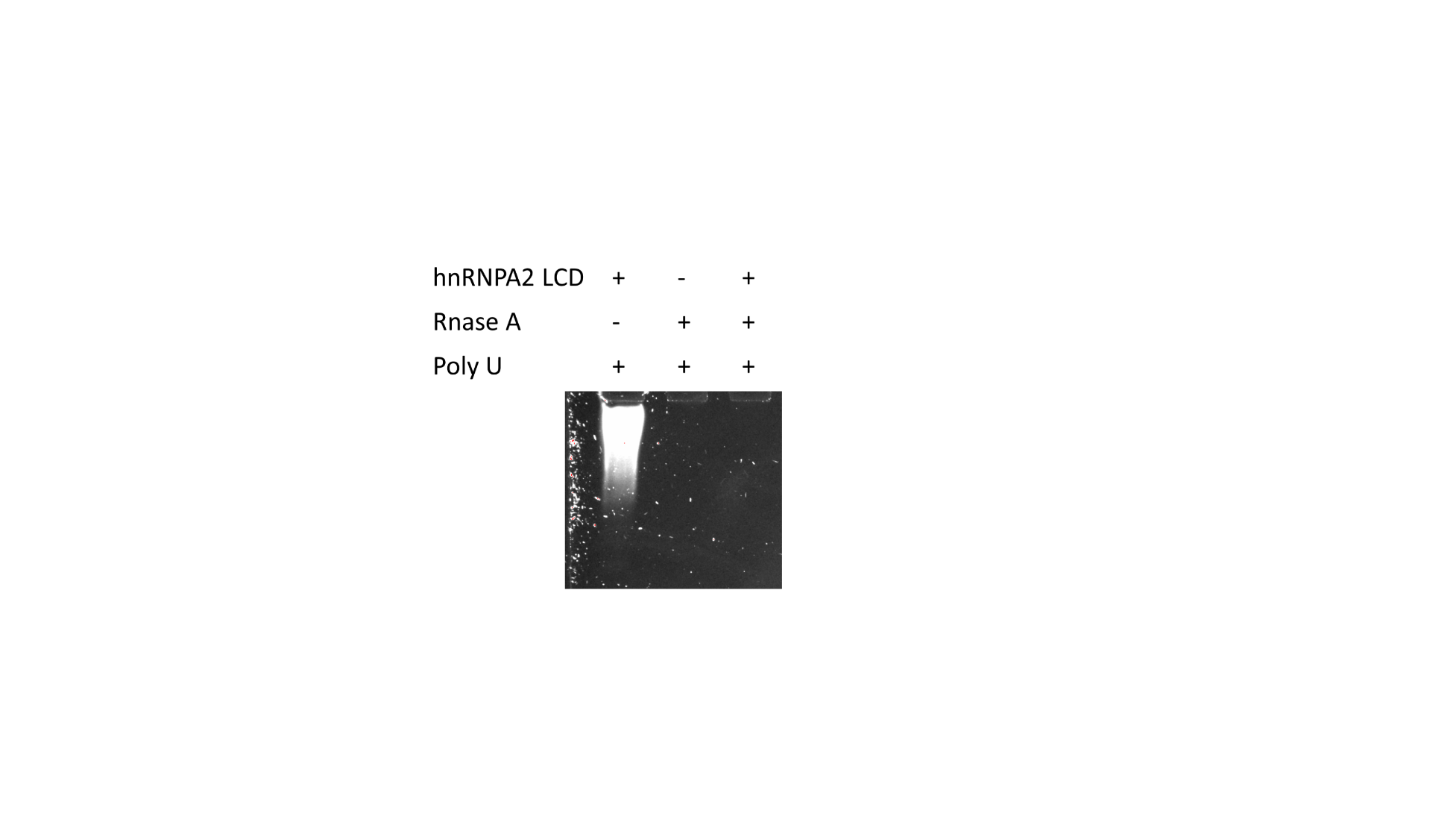


**Supplementary Figure 3**10% Native PAGE stained with SYBR gold. Upon adding RNAse A, polyU gets degraded, independently of the presence of hnRNPA2 LCD.


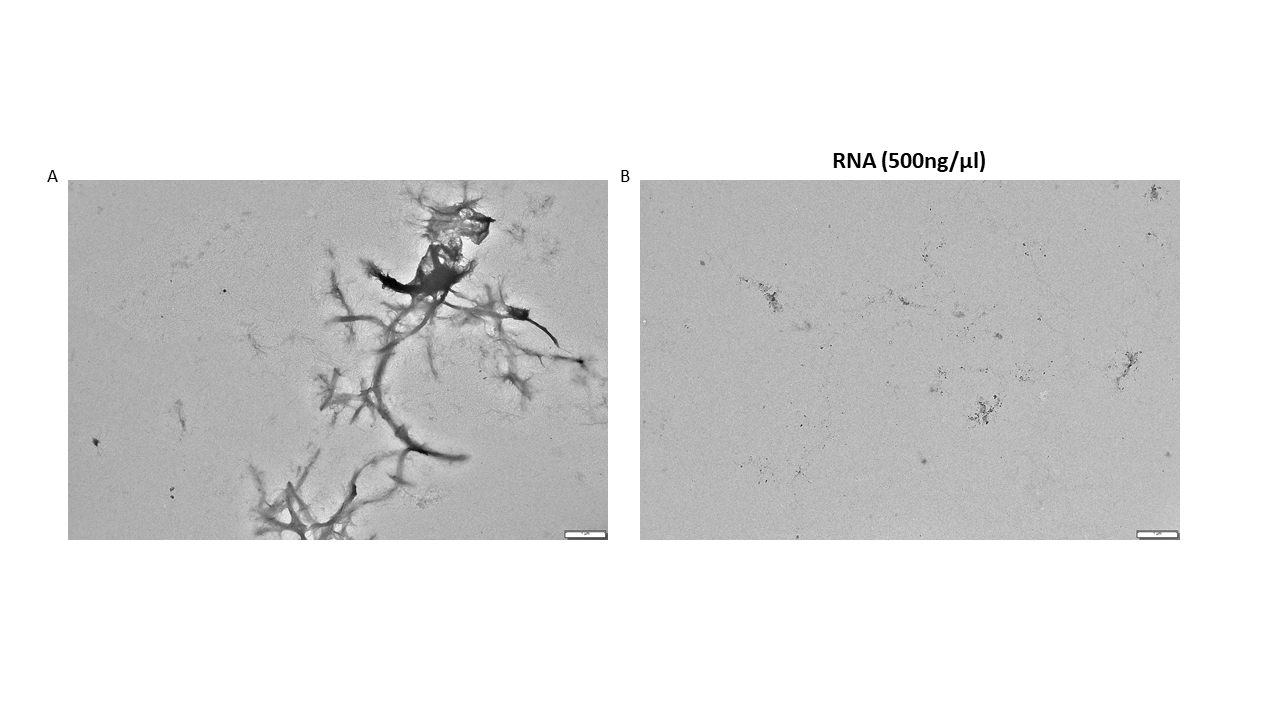


**Supplementary Figure 4**

TEM image of hnRNPA2 LCD D290V fibrils without (**A**) and with (**B**) RNA (polyU).
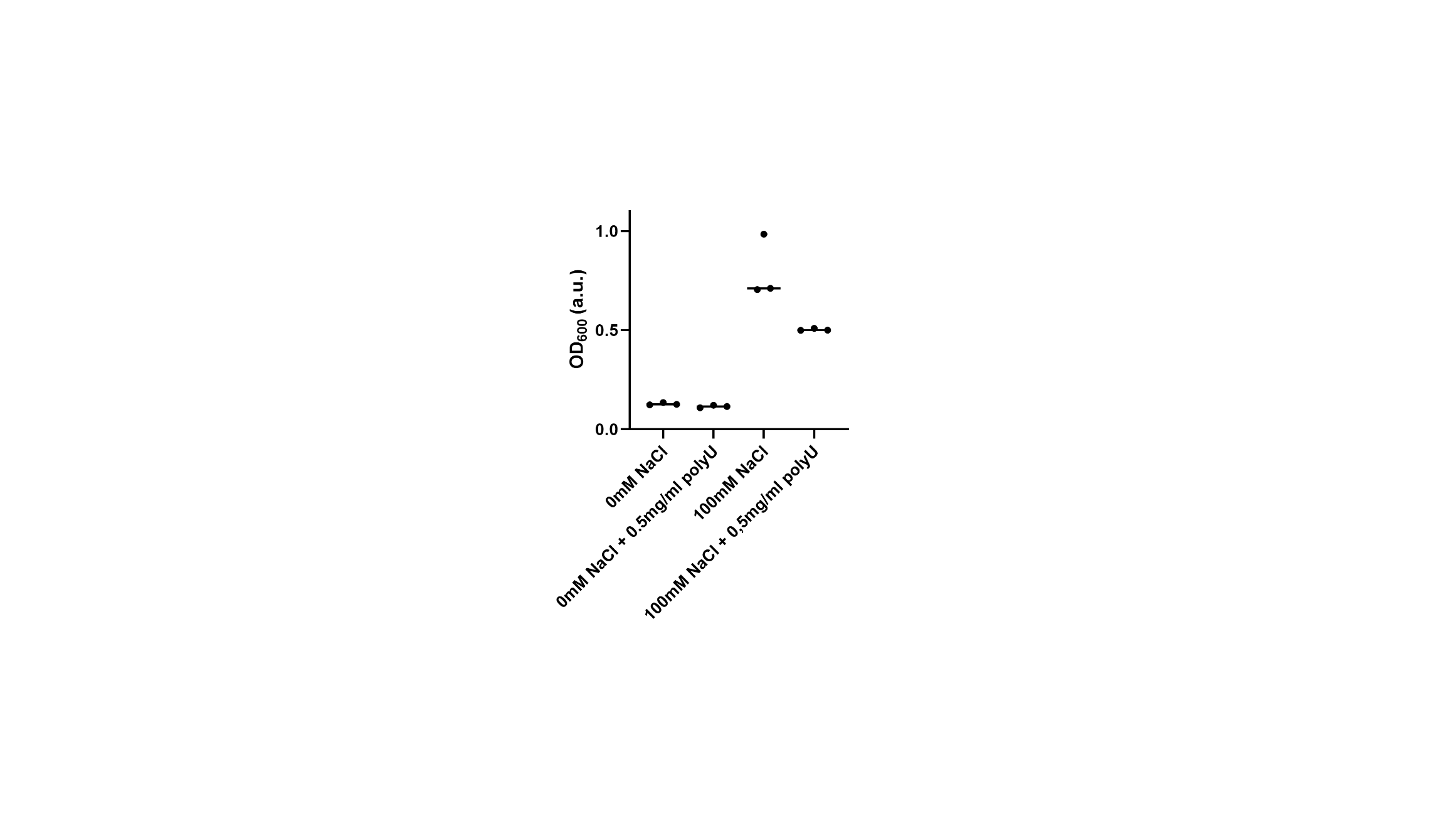


**Supplementary Figure 5**

Turbidity as a readout of the effect of RNA on hnRNPA2 LCD D290V LLPS. Comparable to figure 6, RNA decreased LLPS of hnRNPA2 LCD D290V.


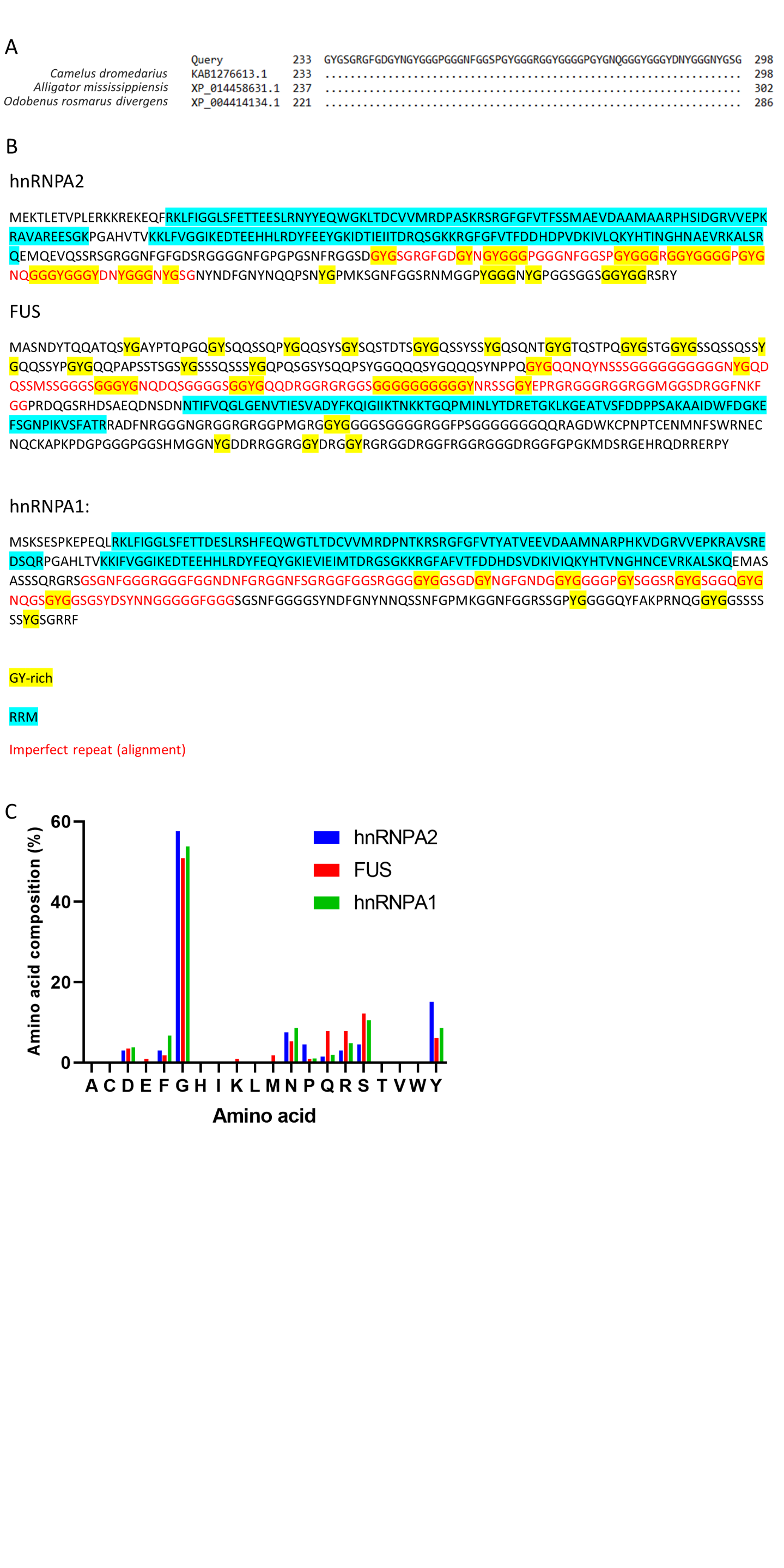


**Supplementary Figure 6**

**(A)** Human hnRNPA2 imperfect repeat sequence was aligned with camel (*Camelus dromedaries*), alligator (*Alligator mississippiensis*) and walrus (*Odobenus rosmarus* divergens) sequences. Dots mean perfect alignment **(B)** hnRPA2, hnRNPA1 and FUS sequences. Blue = RRM, Yellow = GY-patches, imperfect repeat = low complexity domain nucleic acid interaction domain = red. **(C)** Amino acid distribution of the low complexity domain nucleic acid interaction domain of hnRNPA2, hnRNPA1 and FUS.


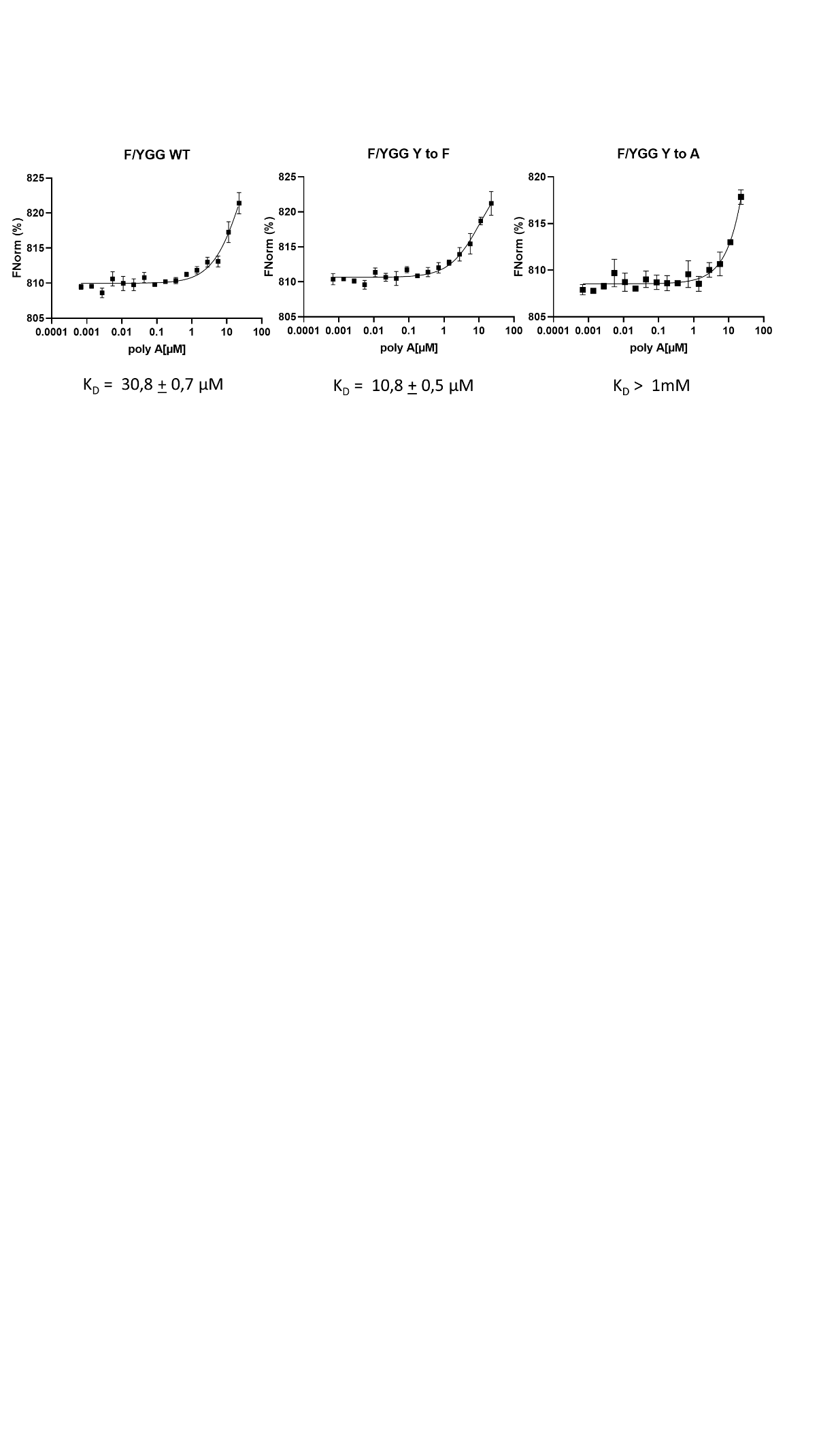


**Supplementary Figure 7**

MST data of F/YGG WT, F/YGG Y to F and F/YGG Y to A peptides , used to calculate the dissociation constants represented in figure 7. Error Bars represent standard deviation from triplicate experiments.


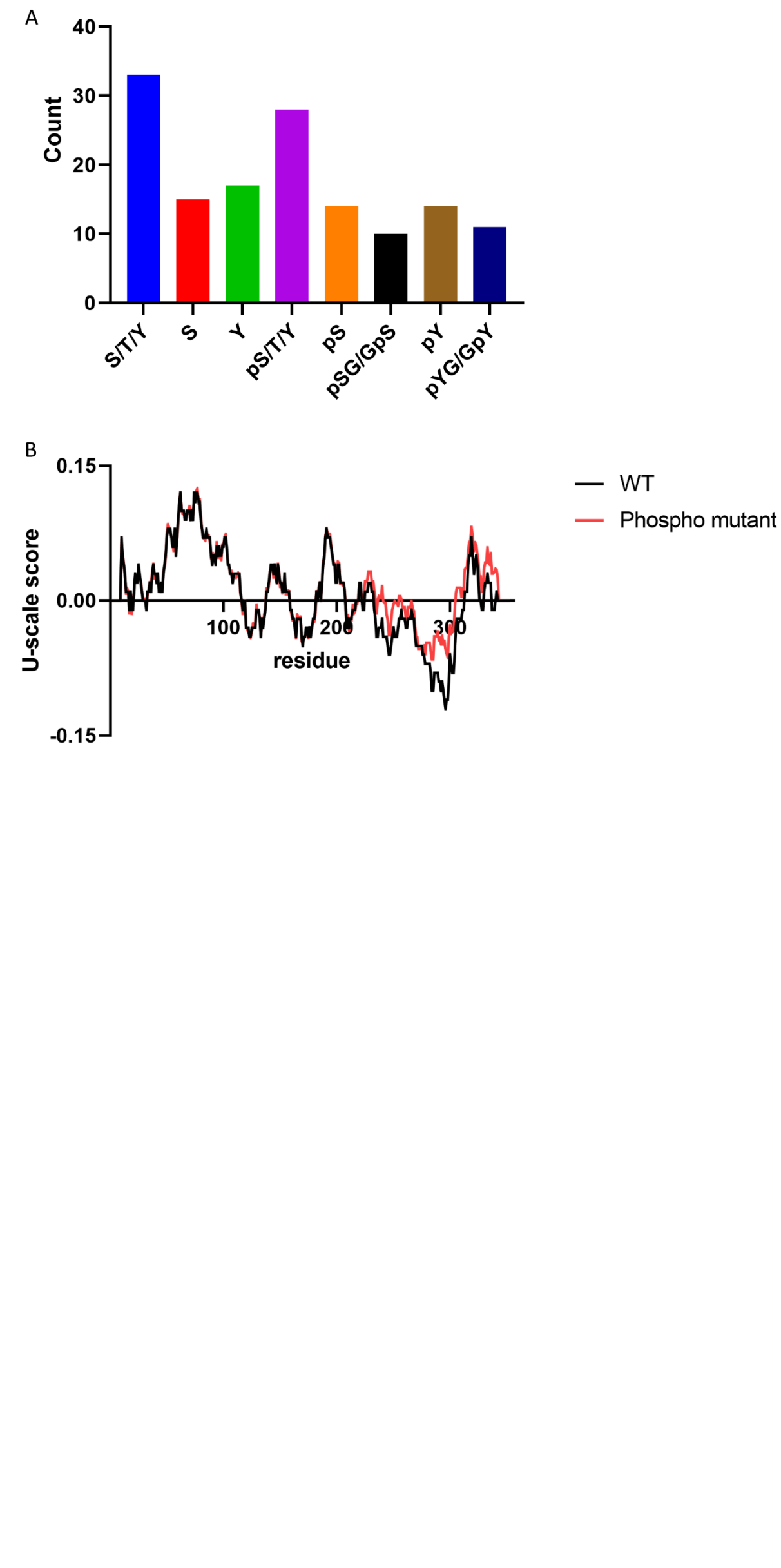


**Supplementary Figure 8**

Multisite phosphorylations as a regulatory mechanism to tune the properties of hnRNPA2’s low-complexity domain (LCD). **(A)** Annotated phosphosites of hnRNPA2’s LCD from PhosphoSitePlus. The figure shows that a very high number of S and Y amino acids are phosphorylated, and these are often flanked by glycines. **(B)** U-scale predicts that hnRNPA2 LCD is sensitive to multisite phosphorylations of Y residues. LCD of the WT protein (black) is predicted to bind RNA (especially polyU) with more favorable free energy compared to the phosphomimetic mutant (all pY sites of the LCD are mutated to E).
